# Perceptions, Trust, and Barriers Related to the Adoption of Artificial Intelligence for Assessing Dental Implant Success and Failure Among Dental Professionals: A Cross-Sectional Survey

**DOI:** 10.7759/cureus.115669

**Published:** 2026-09-02

**Authors:** Pritha Negi, Adeeb Hasan, Tejinderjit Kaur, Komal Thakkar, Navya Yeravelli, Sreelatha Sadasivan, Mazen A Almasri

**Affiliations:** 1 Department of Prosthodontics, Crown and Bridge, K. M. Shah Dental College and Hospital, Sumandeep Vidyapeeth (Deemed to be University), Vadodara, IND; 2 Department of Oral and Maxillofacial Surgery, Sri Sai Dental College and Research Institute, Srikakulam, IND; 3 Department of Dentistry, Guru Gobind Singh Medical College and Hospital, Faridkot, IND; 4 Department of Periodontology, Pacific Dental College and Research Centre, Udaipur, IND; 5 Department of Information Technology, University of the Cumberlands, Williamsburg, USA; 6 Department of Oral and Maxillofacial Surgery, Sri Sai College of Dental Surgery, Vikarabad, IND; 7 Department of Oral Maxillofacial Surgery, King Abdulaziz University, Jeddah, SAU

**Keywords:** artificial intelligence, dental implants, dental professionals, surveys and questionnaires, technology adoption

## Abstract

Introduction

Artificial intelligence (AI) is increasingly being explored in implant dentistry to improve diagnosis, treatment planning, and the prediction of implant success or failure. However, successful clinical implementation depends largely on dental professionals’ awareness, trust, and acceptance. This study aimed to evaluate the awareness, knowledge, perceptions, trust, acceptance, and barriers to the adoption of AI for assessing dental implant success and failure among dental professionals.

Materials and methods

A questionnaire-based cross-sectional survey was conducted among 450 dental professionals, comprising 150 practitioners, 150 academicians, and 150 trainees. The validated questionnaire contained 15 items covering four domains: demographic characteristics, awareness and knowledge, perceptions and attitudes, and trust and acceptance of AI. Descriptive statistics were calculated, and intergroup comparisons were performed using the chi-square and Kruskal-Wallis tests. Mann-Whitney U tests, Jonckheere-Terpstra trend analysis, and multivariable ordinal logistic regression were performed where appropriate. Statistical significance was set at p < 0.05.

Results

Significant differences in AI awareness and acceptance were observed among the professional groups. Awareness of AI applications in implant assessment was highest among academicians, followed by trainees and practitioners (p < 0.001). High or complete trust in AI-generated implant predictions was reported by 85 (56.7%) academicians, 70 (46.7%) trainees, and 40 (26.7%) practitioners. Similarly, willingness to incorporate AI into routine clinical practice was significantly greater among trainees and academicians than among practitioners (p < 0.001). Lack of training and education was the most frequently reported barrier, whereas practitioners primarily identified implementation costs, and academicians highlighted ethical and medicolegal concerns. Multivariable ordinal logistic regression demonstrated that trainee status (odds ratio (OR) = 7.03), academic status (OR = 3.49), greater AI familiarity (OR = 2.27), and higher trust (OR = 2.59) were significant positive predictors of AI adoption, whereas increasing age (OR = 0.64) and years of experience (OR = 0.92) were negatively associated with willingness to adopt AI.

Conclusions

Dental professionals demonstrated significant differences in their awareness, trust, and acceptance of AI for implant success and failure assessment. Greater familiarity and trust were associated with greater willingness to adopt AI, whereas older age and greater clinical experience were associated with lower acceptance. Structured educational initiatives, clinical validation, and appropriate regulatory frameworks may facilitate the integration of AI into implant dentistry.

## Introduction

Artificial intelligence (AI) has emerged as one of the most transformative technologies in modern healthcare, with applications ranging from disease diagnosis and treatment planning to prognostic prediction and personalized patient care [1]. In dentistry, AI has demonstrated promising capabilities in image interpretation, automated diagnosis, orthodontic treatment planning, caries detection, periodontal assessment, and oral pathology [2,3]. More recently, AI-driven algorithms have been developed to support implant dentistry by analyzing radiographic and clinical datasets to predict implant success, estimate failure risk, assess peri-implant bone changes, and assist practitioners in evidence-based decision-making [4]. These advancements have the potential to improve diagnostic consistency, optimize treatment outcomes, and enhance the efficiency of implant care.

Despite these technological developments, the successful integration of AI into routine implant practice depends not only on algorithmic performance but also on the willingness of dental professionals to adopt and trust these systems [5]. Clinical acceptance is influenced by several factors, including familiarity with AI technologies, perceived usefulness, confidence in prediction accuracy, ethical and medicolegal considerations, accessibility of AI-enabled systems, implementation costs, and concerns regarding the role of AI in clinical decision-making [6,7]. Although AI is intended to augment rather than replace professional expertise, apprehensions regarding reliability, transparency, accountability, and the potential erosion of clinical autonomy continue to influence practitioner attitudes [8]. Furthermore, differences in educational background, years of professional experience, and academic exposure may contribute to variations in awareness and acceptance among practitioners, academicians, and trainees [7]. Understanding these differences is essential for designing targeted educational initiatives and implementation strategies to facilitate the integration of AI into implant dentistry.

Current literature has primarily focused on the technical performance of AI models in implant diagnosis and prognosis, whereas comparatively limited attention has been directed toward evaluating the perceptions and readiness of dental professionals to incorporate these technologies into clinical practice [4,9]. Assessing awareness, trust, acceptance, and perceived barriers among different professional groups can provide valuable insights for curriculum development, continuing professional education, policy formulation, and the future clinical implementation of AI-assisted implant care.

Therefore, the present cross-sectional survey aimed to assess the awareness, perceptions, trust, and willingness of dental professionals to adopt AI for dental implant success and failure assessment. The primary objective was to evaluate these domains among practitioners, academicians, and trainees. The secondary objectives were to identify perceived barriers to AI adoption and determine demographic and professional factors, including professional designation, associated with willingness to adopt AI. Comparison among the three professional groups was undertaken because differences in clinical experience, academic exposure, and familiarity with emerging technologies may influence AI awareness, trust, and acceptance.

## Materials and methods

Study design and setting

This questionnaire-based cross-sectional survey was conducted in the Department of Oral and Maxillofacial Surgery, Sri Sai College of Dental Surgery, Vikarabad, India, over six months, from July to December 2023. The study protocol was reviewed and approved by the Institutional Ethics Committee (52/SSCDS/Acad/Ethical/2023) before participant recruitment. All procedures were performed in accordance with the ethical principles outlined in the Declaration of Helsinki (2013 revision). Participation was voluntary, and electronic informed consent was obtained from all respondents before they commenced the survey.

Study population and sampling

This study included dental professionals recruited from various academic institutions and private clinical practices across India. Participants comprised three predefined professional groups: practicing dentists, academicians involved in undergraduate or postgraduate dental education, and postgraduate trainees/interns pursuing dental specialization. Eligible participants were registered dental professionals aged 25 years or older who were actively involved in clinical practice, dental education, or postgraduate training and were willing to provide informed consent. Individuals who were not registered as dental professionals, undergraduate students, retired dentists not engaged in active practice, respondents providing incomplete questionnaires (> 20% missing responses), or duplicate submissions were excluded from the study.

A non-probability purposive sampling approach with predefined professional-group quotas was used to ensure adequate representation of the three professional categories. A target of 150 participants was established for each group, resulting in a total sample of 450 participants. Recruitment was continued within each professional category until the prespecified quota of 150 eligible respondents was reached.

Potential participants were approached through dental institutions, professional contacts, and clinical and postgraduate training settings. The survey was distributed electronically, and participation was voluntary. Before accessing the questionnaire, all respondents were provided with information regarding the study and gave electronic informed consent. Eligibility was verified based on professional status and the predefined inclusion and exclusion criteria. Participants were enrolled only once, and duplicate submissions were excluded. Respondents with incomplete questionnaires (> 20% missing responses) were excluded from the final analysis. This quota-based approach was adopted to facilitate meaningful comparisons among practitioners, academicians, and trainees.

Questionnaire development

A structured self-administered questionnaire was specifically developed for this study following an extensive review of the published literature on AI in dentistry, implant dentistry, technology acceptance models, and healthcare AI adoption. The questionnaire was developed by a multidisciplinary expert panel comprising an AI specialist with expertise in healthcare applications, oral and maxillofacial surgeons with experience in implant dentistry, periodontists, prosthodontists, oral radiologists, and biostatisticians. The expert panel ensured that the questionnaire comprehensively addressed the study objectives while maintaining scientific validity and clinical relevance. Initially, an item pool consisting of 28 questions was created. Following expert review, redundant and ambiguous items were removed, resulting in a final questionnaire containing 15 items divided into four major sections (Appendices).

Content validation and pilot testing

The preliminary questionnaire underwent content validation by an expert panel. Each item was evaluated for clarity, relevance, comprehensiveness, and simplicity using a four-point rating scale. Based on the experts' recommendations, minor modifications were made to improve wording and readability. A pilot study was subsequently conducted with 30 dental professionals who were not included in the final study sample. Feedback obtained during pilot testing was used to refine the sequence of questions, simplify the language where necessary, and estimate the average time required to complete the questionnaire, which was approximately 8-10 minutes.

Reliability testing and calibration

Before the commencement of the main survey, all investigators involved in questionnaire administration underwent calibration sessions to standardize participant instructions, informed consent procedures, and methods of questionnaire distribution. Pilot responses were used to assess the psychometric properties of the questionnaire. The internal consistency reliability of the perception domain was evaluated using Cronbach's alpha coefficient. The questionnaire demonstrated good internal consistency, with a Cronbach's alpha of 0.82, indicating acceptable reliability for subsequent analyses.

Questionnaire structure and scoring

The final questionnaire comprised 15 items organized into four thematic sections and was designed to comprehensively evaluate dental professionals' awareness, knowledge, perceptions, trust, acceptance, and perceived barriers regarding the use of AI in dental implant success and failure assessment (Appendices). The first section collected demographic and professional information, including age, sex, professional designation, and years of professional experience. The second section assessed participants' awareness and knowledge of AI in implant dentistry by evaluating their familiarity with AI, awareness of its applications in implant assessment, and knowledge of its potential clinical uses. The third section evaluated participants' perceptions and attitudes toward AI using statements related to its ability to predict implant success and failure, the consistency of AI-based assessments with human clinical judgment, and its role as a decision-support system. The final section explored respondents' trust in AI-generated predictions, willingness to incorporate AI into clinical practice, perceived barriers to AI adoption, and their views on including AI education in dental curricula and continuing professional development programs.

Responses to perception, trust, and acceptance-related items were recorded using five-point Likert-type or ordinal rating scales, with higher scores representing greater familiarity, more favorable perceptions, stronger trust, and greater willingness to adopt AI in implant dentistry. Questions assessing knowledge of AI applications and barriers to adoption employed multiple-response formats, allowing participants to select all applicable options or up to three responses, as appropriate. Demographic variables and categorical responses were coded numerically before statistical analysis. Individual item scores were analyzed independently rather than combined into an overall composite score, enabling domain-specific assessment of awareness, perceptions, trust, and acceptance among the three professional groups.

Sample size estimation

The sample size was estimated using the Calculator.net online sample size calculator [10]. Assuming an infinite population, a 95% confidence level, a 5% margin of error, and a conservative response distribution of 50%, the minimum required sample size was calculated as 384 participants. To compensate for incomplete responses, non-responses, and exclusions during data cleaning (approximately 15%), the sample size was increased. Furthermore, to facilitate robust subgroup comparisons among practitioners, academicians, and trainees, a target of 150 participants was established for each professional category, resulting in a final target sample size of 450 participants.

Survey distribution and response enhancement

The finalized questionnaire was converted into an electronic survey using Google Forms. The survey link was distributed through professional dental networks, including email and WhatsApp groups of dental practitioners, academicians, and postgraduate trainees from various institutions and private practices across India. Participation was voluntary, and each participant could submit only one response. The first page of the survey contained the participant information sheet and an electronic informed consent statement, and respondents could proceed only after providing consent. The questionnaire completion time was approximately 8-10 minutes based on pilot testing.

To enhance the response rate, reminder messages were sent at two-week intervals to eligible participants who had not yet responded. The questionnaire was designed to require approximately 8-10 minutes for completion, with clear and concise questions to minimize respondent burden. Participation was anonymous, no financial incentives were provided, and confidentiality of responses was assured to encourage honest participation. Duplicate submissions were prevented using Google Forms settings, and incomplete questionnaires (> 20% missing responses) were excluded from the final analysis.

Statistical analysis

Data were analyzed using IBM SPSS Statistics for Windows (version 25.0; IBM Corp., Armonk, NY). Descriptive statistics are expressed as frequencies and percentages for categorical variables and as means with standard deviations or medians with interquartile ranges for continuous and ordinal variables, as appropriate. Comparisons of categorical variables among the three professional groups were performed using the chi-squared test. Differences in ordinal perception and attitude scores were analyzed using the Kruskal-Wallis H test, followed by Bonferroni-adjusted Mann-Whitney U tests for pairwise comparisons. The Jonckheere-Terpstra test was employed to evaluate monotonic trends in trust across professional groups. The internal consistency of the perception scale was assessed using Cronbach's alpha. Multivariable ordinal logistic regression (proportional odds model) was performed to identify independent predictors of willingness to adopt AI in implant dentistry. Model performance was evaluated using the Nagelkerke pseudo R², the test of parallel lines, and the Hosmer-Lemeshow goodness-of-fit test. Effect sizes were reported using eta squared (η²) for Kruskal-Wallis analyses and Cramér's V for chi-squared analyses. All statistical tests were two-tailed, and statistical significance was set at p < 0.05.

## Results

A total of 450 dental professionals, comprising 150 practitioners, 150 academicians, and 150 trainees, completed the survey. Significant differences were observed among the three professional groups in terms of age distribution and years of professional experience (both p < 0.001), whereas the sex distribution was comparable across the groups (p = 0.504). Overall, 255 (56.7%) participants were male, and 195 (43.3%) were female. The majority of the trainees belonged to the 25-34 years age group and had less than five years of professional experience (Table 1).

**Table 1:** Demographic and professional characteristics of the study participants by professional group ^*^P < 0.05 was considered statistically significant Comparisons between professional groups were performed using the Pearson chi-square (χ²) test df: degrees of freedom; χ²: chi-square statistic

Characteristic	Category	Practitioners (n = 150), n (%)	Academicians (n = 150), n (%)	Trainees (n = 150), n (%)	Total (N = 450), n (%)	Test statistic	P-value
Age group, years	25–34	15 (10.0%)	30 (20.0%)	120 (80.0%)	165 (36.7%)	χ² = 162.4 (df = 6)	< 0.001^*^
	35–44	45 (30.0%)	50 (33.3%)	20 (13.3%)	115 (25.6%)		
	45–54	60 (40.0%)	45 (30.0%)	8 (5.3%)	113 (25.1%)		
	≥ 55	30 (20.0%)	25 (16.7%)	2 (1.3%)	57 (12.7%)		
Sex	Male	90 (60.0%)	85 (56.7%)	80 (53.3%)	255 (56.7%)	χ² = 1.37 (df = 2)	0.504
	Female	60 (40.0%)	65 (43.3%)	70 (46.7%)	195 (43.3%)		
Experience, years	< 5	10 (6.7%)	20 (13.3%)	135 (90.0%)	165 (36.7%)	χ² = 245.3 (df = 6)	< 0.001^*^
	5–10	40 (26.7%)	45 (30.0%)	15 (10.0%)	100 (22.2%)		
	11–20	60 (40.0%)	50 (33.3%)	0 (0.0%)	110 (24.4%)		
	> 20	40 (26.7%)	35 (23.3%)	0 (0.0%)	75 (16.7%)		

Academicians demonstrated the highest familiarity with AI (mean: 4.0 ± 0.9), followed by trainees (3.5 ± 1.0) and practitioners (2.6 ± 1.1) (p < 0.001). Awareness of AI applications for implant assessment was reported by 135 (90.0%) academicians, 120 (80.0%) trainees, and 85 (56.7%) practitioners (p < 0.001), as shown in Table 2.

**Table 2:** Awareness and familiarity with artificial intelligence applications in implant assessment by professional group ^*^P < 0.05 was considered statistically significant Kruskal-Wallis test was used for comparisons of continuous variables, and the Pearson chi-square (χ²) test was used for categorical variables; effect size is reported as eta squared (η²) for continuous variables and Cramér's V for categorical variables SD: standard deviation; IQR: interquartile range; η²: eta squared; χ²: chi-square statistic

Variable	Category	Practitioners	Academicians	Trainees	Test	P-value	Effect size
Q5: Familiarity	Mean ± SD	2.6 ± 1.1	4.0 ± 0.9	3.5 ± 1.0	H = 94.8	< 0.001^*^	Eta² = 0.21
	Median (IQR)	3.0 (2.0–3.0)	4.0 (3.0–5.0)	4.0 (3.0–4.0)			
Q6: Awareness of AI in implant assessment, n (%)	Yes	85 (56.7%)	135 (90.0%)	120 (80.0%)	χ² = 44.1	< 0.001^*^	Cramér’s V = 0.221
	No	40 (26.7%)	8 (5.3%)	15 (10.0%)			
	Unsure	25 (16.7%)	7 (4.7%)	15 (10.0%)			

Implant treatment planning was the most frequently recognized application, reported by 145 (96.7%) academicians, 140 (93.3%) trainees, and 120 (80.0%) practitioners. Similarly, 140 (93.3%) academicians, 130 (86.7%) trainees, and 95 (63.3%) practitioners reported using AI to predict implant success or failure. Significant intergroup differences were observed for all the listed AI applications (p ≤ 0.002) (Table 3).

**Table 3:** Knowledge of artificial intelligence applications in implant dentistry by professional group ^*^P < 0.05 was considered statistically significant Comparisons between professional groups were performed using the Pearson chi-square (χ²) test; effect size is presented as Cramér's V χ²: chi-square statistic

Q7: Perceived applications (multiple select)	Practitioners, n (%)	Academicians, n (%)	Trainees, n (%)	Test statistics (χ²)	P-value	Effect size: Cramér’s V
Implant identification from radiographs	110 (73.3%)	140 (93.3%)	135 (90.0%)	26.6	< 0.001^*^	0.172
Implant treatment planning	120 (80.0%)	145 (96.7%)	140 (93.3%)	24.0	< 0.001^*^	0.163
Predicting success/failure outcomes	95 (63.3%)	140 (93.3%)	130 (86.7%)	45.7	< 0.001^*^	0.225
Assessing peri-implant bone loss	85 (56.7%)	130 (86.7%)	110 (73.3%)	33.4	< 0.001^*^	0.193
Monitoring osseointegration	75 (50.0%)	120 (80.0%)	125 (83.3%)	46.5	< 0.001^*^	0.228
No application	15 (10.0%)	2 (1.3%)	5 (3.3%)	12.7	0.002^*^	0.119

Academicians and trainees expressed significantly greater agreement regarding AI's ability to predict implant success (mean: 4.2 ± 0.8 and 4.0 ± 0.9, respectively) compared with practitioners (mean: 3.2 ± 1.1) (p < 0.001). Likewise, practitioners were less likely to believe that AI could provide more consistent assessments than human clinical judgment (mean: 2.8 ± 1.2) compared with academicians (mean: 3.8 ± 1.0) and trainees (mean: 3.9 ± 0.9) (p < 0.001). Nevertheless, all three groups strongly agreed that AI should function as a decision-support tool rather than replace clinical judgment, with mean scores ranging from 4.4 to 4.6 (Table 4).

**Table 4:** Perceptions and attitudes toward artificial intelligence in implant dentistry by professional group ^*^P < 0.05 was considered statistically significant Comparisons between professional groups were performed using the Kruskal-Wallis test SD: standard deviation; IQR: interquartile range; df: degrees of freedom

Item	Group	Mean ± SD	Median (IQR)	Kruskal-Wallis (H, df = 2)	P-value	Effect size
Q8: Predict success	Practitioners	3.2 ± 1.1	3.0 (2.0–4.0)	68.4	< 0.001^*^	0.19
	Academicians	4.2 ± 0.8	4.0 (4.0–5.0)			
	Trainees	4.0 ± 0.9	4.0 (3.0–5.0)			
Q9: Predict failure	Practitioners	3.0 ± 1.0	3.0 (2.0–4.0)	52.1	< 0.001^*^	0.18
	Academicians	4.0 ± 0.9	4.0 (3.0–5.0)			
	Trainees	3.8 ± 1.0	4.0 (3.0–4.0)			
Q10: More consistent than humans	Practitioners	2.8 ± 1.2	3.0 (2.0–4.0)	72.0	< 0.001^*^	0.16
	Academicians	3.8 ± 1.0	4.0 (3.0–5.0)			
	Trainees	3.9 ± 0.9	4.0 (3.0–5.0)			
Q11: Decision-support (not replace)	Practitioners	4.5 ± 0.7	5.0 (4.0–5.0)	8.5	0.014^*^	0.12
	Academicians	4.6 ± 0.6	5.0 (4.0–5.0)			
	Trainees	4.4 ± 0.8	5.0 (4.0–5.0)			

High or complete trust in AI-generated implant predictions was reported by 85 (56.7%) academicians, 70 (46.7%) trainees, and 40 (26.7%) practitioners (p < 0.001). Similarly, willingness to incorporate AI into routine implant practice was highest among trainees (120 (80.0%)), followed by academicians (100 (66.7%)) and practitioners (55 (36.7%)) (p < 0.001). The Jonckheere-Terpstra test demonstrated a significant increasing trend in trust across professional groups (p < 0.001), as shown in Table 5.

**Table 5:** Trust in and acceptance of artificial intelligence systems by professional group *P < 0.05 was considered statistically significant Group comparisons were performed using the Pearson chi-square (χ²) test, whereas ordinal trend analysis was performed using the Jonckheere–Terpstra test; effect size is reported as Cramér's V for categorical comparisons AI: artificial intelligence; SD: standard deviation; χ²: chi-square statistic

Variable	Category	Practitioners	Academicians	Trainees	Test	P-value	Effect size
Q12: Trust in AI predictions (Ordinal 1–5)	No/low trust, n (%)	60 (40.0%)	25 (16.7%)	35 (23.3%)	χ² = 33.51	< 0.001^*^	Cramér’s V = 0.192
	Moderate trust, n (%)	50 (33.3%)	40 (26.7%)	45 (30.0%)			
	High/complete trust, n (%)	40 (26.7%)	85 (56.7%)	70 (46.7%)			
	Mean ± SD	2.80 ± 1.10	3.53 ± 1.02	3.40 ± 1.05	z = 6.82	< 0.001^*^	0.08
Q13: Willingness to incorporate	Yes (Def/Prob), n (%)	55 (36.7%)	100 (66.7%)	120 (80.0%)	χ² = 66.8	< 0.001^*^	Cramér’s V = 0.272
	Uncertain, n (%)	45 (30.0%)	30 (20.0%)	20 (13.3%)			
	Probably/Def not, n (%)	50 (33.3%)	20 (13.3%)	10 (6.7%)			

Lack of training and education was the most frequently reported barrier, particularly among trainees. In contrast, practitioners most commonly identified the high cost of implementation, whereas academicians predominantly reported ethical and medicolegal concerns. Most of the barriers differed significantly among the three professional groups (p < 0.001), whereas concerns regarding reliability and errors (p = 0.116) and replacing human roles with AI (p = 0.067) did not demonstrate statistically significant differences (Table 6).

**Table 6:** Primary barriers to the adoption of artificial intelligence in implant dentistry by professional group ^*^P < 0.05 was considered statistically significant As multiple responses were permitted, percentages do not sum to 100%; comparisons for each barrier between professional groups were performed using the Pearson chi-square (χ²) test χ²: chi-square statistic; AI: artificial intelligence

Q 14: Barriers (select up to 3)	Practitioners (n = 150), n (%)	Academicians (n = 150), n (%)	Trainees (n = 150), n (%)	Total (n = 450), n (%)	χ² test (per barrier)	P-value
Lack of training and education	40 (26.7%)	60 (40.0%)	120 (80.0%)	220 (48.9%)	97.3	< 0.001^*^
High cost of implementation	90 (60.0%)	45 (30.0%)	50 (33.3%)	185 (41.1%)	37.1	< 0.001^*^
Insufficient evidence on AI accuracy	50 (33.3%)	70 (46.7%)	40 (26.7%)	160 (35.6%)	13.5	0.001^*^
Concerns about reliability and errors	70 (46.7%)	55 (36.7%)	70 (46.7%)	195 (43.3%)	4.3	0.116
Ethical and medico-legal concerns	40 (26.7%)	80 (53.3%)	45 (30.0%)	165 (36.7%)	29.0	< 0.001^*^
Lack of access to AI-integrated systems	75 (50.0%)	40 (26.7%)	55 (36.7%)	170 (37.8%)	18.2	< 0.001^*^
Resistance to change	45 (30.0%)	25 (16.7%)	15 (10.0%)	85 (18.9%)	20.4	< 0.001^*^
Concerns about AI replacing human roles	30 (20.0%)	35 (23.3%)	20 (13.3%)	85 (18.9%)	5.4	0.067

Compared with practitioners, trainees (OR = 7.03; 95% CI: 3.83-12.91; p < 0.001) and academicians (OR = 3.49; 95% CI: 2.02-6.03; p < 0.001) exhibited significantly greater willingness to adopt AI. Higher familiarity with AI (OR = 2.27; p < 0.001) and greater trust in AI (OR = 2.59; p < 0.001) were also significant positive predictors of AI adoption, whereas increasing age (OR = 0.64; p = 0.012) and years of professional experience (OR = 0.92; p = 0.046) were negatively associated with willingness to adopt AI. A Nagelkerke pseudo R² of 0.482 indicated that the model explained approximately 48.2% of the variance in willingness to adopt AI. A nonsignificant test of parallel lines (χ² = 12.4, p = 0.19) confirmed that the proportional odds assumption was satisfied. A nonsignificant Hosmer-Lemeshow goodness-of-fit test (χ² = 8.1, p = 0.42) indicated an adequate model fit (Table 7).

**Table 7:** Ordinal logistic regression analysis for predictors of willingness to adopt artificial intelligence in implant dentistry ^*^P < 0.05 was considered statistically significant Multivariable ordinal logistic regression was used to identify independent predictors of willingness to adopt artificial intelligence β: regression coefficient; SE: standard error; χ²: Wald chi-square statistic; OR: odds ratio; CI: confidence interval; Ref: reference category; AI: artificial intelligence

Predictor		β coefficient	SE	Wald χ²	P-value	OR	95% CI for OR
Age group (Ref: ≥ 55)		-0.45	0.18	6.25	0.012^*^	0.64	(0.45–0.91)
Sex (Ref: Male)		0.12	0.22	0.30	0.584	1.13	(0.73–1.74)
Professional group (Ref: practitioners)	Academicians	1.25	0.28	19.9	< 0.001^*^	3.49	(2.02–6.03)
	Trainees	1.95	0.31	39.6	< 0.001^*^	7.03	(3.83–12.91)
Experience (years, continuous)		-0.08	0.04	4.00	0.046^*^	0.92	(0.85–0.99)
Q5: AI familiarity (1–5)		0.82	0.12	46.7	< 0.001^*^	2.27	(1.79–2.88)
Q11: Decision-support (1–5)		0.31	0.15	4.27	0.039^*^	1.36	(1.01–1.83)
Q12: Trust score (1–5)		0.95	0.14	46.0	< 0.001^*^	2.59	(1.97–3.41)

Figure 1 demonstrates that the level of support for incorporating AI education into dental curricula and continuing professional development programs varied across the three professional groups. Trainees demonstrated the highest support, with 110 (73%) strongly supporting and 5 (10%) supporting the initiative. Similarly, academicians showed high acceptance, with 97 (65%) strongly supporting and 23 (15%) supporting AI education. In contrast, practitioners exhibited relatively lower support, with 34 (23%) strongly supporting and 40 (27%) supporting, while 26 (17%) opposed and 15 (10%) strongly opposed the proposal.

**Figure 1:**
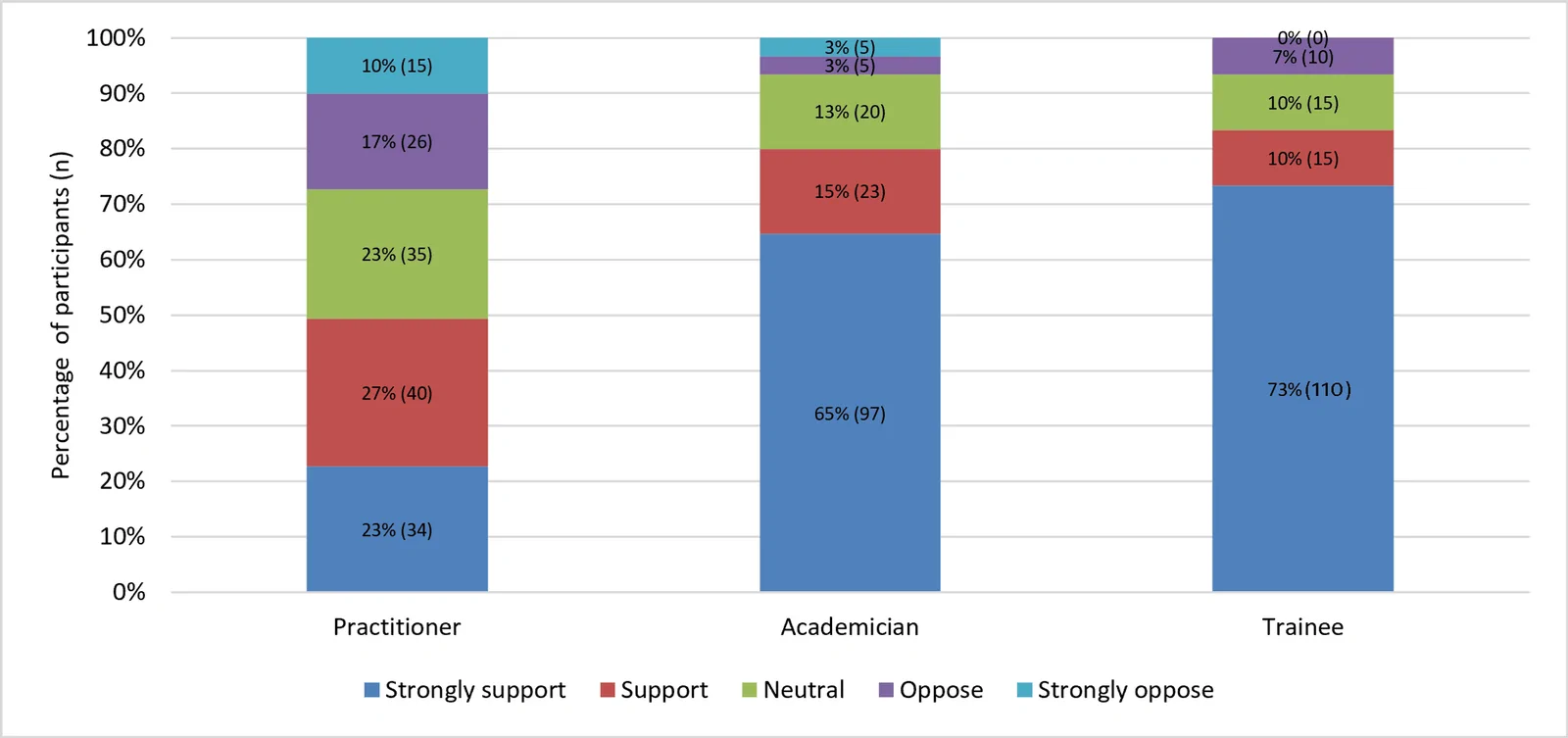
Support for AI education in dental curricula and continuing professional development programs AI: artificial intelligence

## Discussion

This cross-sectional survey evaluated the awareness, knowledge, perceptions, trust, and acceptance of AI for dental implant success and failure assessment among dental professionals. The findings demonstrated a clear professional gradient in AI adoption, with academicians and trainees exhibiting significantly greater familiarity, trust, and willingness to incorporate AI into implant practices than practitioners. These differences were further confirmed by multivariate regression analysis, which identified professional designation, familiarity with AI, and trust in AI as positive predictors of adoption, whereas increasing age and professional experience negatively influenced acceptance. These findings suggest that successful implementation of AI in implant dentistry requires not only technological advancement, but also targeted educational strategies to enhance practitioners' confidence in AI-assisted decision-making [9].

The awareness of AI applications was significantly higher among academicians and trainees than among practitioners. This observation is consistent with the findings of Scheetz et al. [11] and Schwendicke et al. [12], who reported that dental professionals working in academic environments demonstrated greater exposure to emerging digital technologies through research activities, continuing education programs, and postgraduate curricula. Younger practitioners and postgraduate trainees are also more likely to encounter AI-assisted software during their training, which may explain their higher familiarity scores [13]. In contrast, experienced practitioners often rely on established clinical protocols and may have fewer opportunities for structured AI training, which may contribute to their relatively lower awareness.

Knowledge regarding the potential applications of AI was highest for implant treatment planning and the prediction of implant success or failure. This finding reflects the rapid evolution of machine-learning algorithms capable of analyzing radiographic, clinical, and patient-specific variables to improve implant treatment planning and prognosis [4,9]. Similar observations were reported by Liu et al. [14] and Acerra et al. [15], who demonstrated increasing clinician awareness of AI applications in implant positioning, bone quality assessment, and prognosis prediction. Nevertheless, fewer practitioners recognized AI applications in peri-implant bone loss assessment and osseointegration monitoring, indicating that knowledge regarding advanced AI applications remains incomplete outside academic settings [16].

The perception analysis demonstrated that academicians and trainees expressed greater confidence in AI's ability to predict implant success and failure than practitioners. However, irrespective of the professional group, the respondents strongly agreed that AI should function as a decision-support tool rather than replace clinical judgment. This finding aligns with previous research, which emphasizes that AI should complement clinician expertise rather than independently determine treatment decisions [17,18]. The relatively lower confidence observed among practitioners may reflect concerns regarding algorithm transparency, medicolegal accountability, and limited real-world validation of AI systems across diverse patient populations [19].

Trust remains one of the strongest determinants of technology adoption in healthcare, and this study demonstrated significantly greater trust among academicians and trainees. Importantly, ordinal logistic regression identified trust as one of the strongest independent predictors of the willingness to adopt AI. Similar findings were reported in studies evaluating AI acceptance in dentistry by Bajjad et al. [20] and Mintz and Brodie [21], where practitioners with greater confidence in AI-generated recommendations were substantially more likely to incorporate AI into routine practice. The positive association observed between familiarity and trust suggests that increasing educational exposure may enhance confidence in AI-assisted clinical workflows.

The barrier analysis highlighted the distinct challenges faced by different professional groups. Trainees primarily reported a lack of training and education, indicating the need for formal AI instruction in undergraduate and postgraduate education. Practitioners identified implementation costs as the major obstacle, reflecting financial concerns associated with software acquisition, hardware upgrades, and integration into existing practice management systems. Academicians, in contrast, were more concerned about ethical and medicolegal implications, emphasizing issues related to accountability, patient privacy, algorithm transparency, and regulatory oversight. Similar concerns have been consistently reported in surveys conducted by Singh et al. [22] and Liu et al. [23], suggesting that the successful implementation of AI requires multidisciplinary solutions encompassing education, regulatory guidance, and robust clinical validation.

The regression analysis further demonstrated that increasing age and years of professional experience were negatively associated with AI adoption. These findings support the Technology Acceptance Model, which proposes that familiarity and perceived usefulness strongly influence the adoption of emerging technologies [24]. Younger professionals who have received greater exposure to digital technologies during training may therefore demonstrate greater readiness to integrate AI into routine clinical practice than experienced practitioners who were trained primarily in conventional workflows.

Clinical implications

The findings of the present study indicate that educational initiatives should be tailored to the specific needs of different professional groups. Undergraduate and postgraduate curricula should incorporate structured training in AI applications relevant to implant dentistry, whereas continuing dental education programs should focus on enhancing AI literacy among practitioners. Furthermore, clinically validated, transparent, and user-friendly AI systems integrated with implant planning software may enhance clinician confidence and facilitate evidence-based decision-making while preserving professional judgment.

Limitations

This study has certain limitations. As a questionnaire-based cross-sectional survey, the findings represent participants' perceptions at a single point in time and cannot establish causal relationships. Potential self-reporting, response, and selection biases should also be considered, as participants may have provided socially desirable responses or overestimated their familiarity and acceptance of AI. Individuals with greater interest in AI may have been more likely to respond, and the non-probability sampling with equal group allocation may not reflect the broader dental population. These factors may have influenced the observed estimates and limited the generalizability of the findings. In addition, although participants were recruited from different professional groups, the survey was conducted only among Indian dental professionals, which may limit the generalizability of the findings to other countries or healthcare systems. The equal allocation of participants across practitioners, academicians, and trainees, although advantageous for intergroup comparisons, may not reflect their actual distribution within the broader dental professional population. Therefore, the findings may have limited applicability to the overall dental community. Sampling weights were not applied because the primary objective was to compare the predefined professional groups rather than estimate population-level parameters.

Future recommendations

Future multicenter and multinational studies involving larger and more diverse populations are required to validate and strengthen these findings. Longitudinal studies evaluating changes in AI acceptance following structured educational interventions would provide more robust evidence on the factors influencing AI adoption. Future research should also assess real-world clinical performance, cost-effectiveness, ethical considerations, and patient acceptance of AI-assisted implant assessment systems to facilitate their safe and evidence-based integration into routine implant dentistry.

## Conclusions

The present study demonstrated significant differences in awareness, perceptions, trust, and acceptance of AI for dental implant success and failure assessment among dental professionals. Academicians and trainees exhibited greater familiarity, trust, and willingness to adopt AI than practitioners, while older age and greater professional experience were negatively associated with AI adoption. Greater AI familiarity and trust were also positively associated with willingness to adopt AI. These findings highlight the need for structured AI education, robust clinical validation, and clear ethical and regulatory frameworks to support the responsible and evidence-based integration of AI into implant dentistry.

## Appendices

**Table 8:** Questionnaire for study participants

Section A: Demographic and professional information		
Q1	What is your age group?	25–34 years
		35–44 years
		45–54 years
		55 years or above
Q2	What is your gender?	Male
		Female
Q3	What is your primary professional designation?	Primarily academician
		Primarily practitioner
		Trainee
Q4	How many years of professional experience do you have?	< 5 years
		5–10 years
		11–20 years
		> 20 years
Section B: Awareness and knowledge		
Q5	How would you rate your overall familiarity with artificial intelligence (AI) in dentistry?	Not at all familiar
		Slightly familiar
		Moderately familiar
		Very familiar
		Extremely familiar
Q6	Are you aware of AI applications specifically related to dental implant assessment (e.g., implant identification, prognosis prediction, failure risk assessment)?	Yes
		No
		Unsure
Q7	In which of the following areas do you believe AI can be applied in implant dentistry? (Select all that apply)	Implant identification from radiographs
		Implant treatment planning and positioning
		Predicting implant success/failure outcomes
		Assessing peri-implant bone loss
		Monitoring osseointegration
		I do not believe AI has applications in implant dentistry
Section C: Perceptions and attitudes		
Please indicate your level of agreement with the following statements using the scale: 1 = Strongly Disagree 2 = Disagree 3 = Neutral 4 = Agree 5 = Strongly Agree		
Q8	AI has the potential to accurately predict dental implant success.	
Q9	AI has the potential to accurately predict dental implant failure.	
Q10	AI-based assessments of implant prognosis would be more consistent than human clinical judgment.	
Q11	AI should serve as a decision-support tool rather than replace clinical judgment in implant assessment.	
Section D: Acceptance, trust, and barriers		
Q12	How much trust do you have in AI-generated predictions of dental implant outcomes?	No trust at all
		Low trust
		Moderate trust
		High trust
		Complete trust
Q13	Would you be willing to incorporate AI-based implant prognosis tools into your clinical practice?	Yes, definitely
		Yes, probably
		Uncertain
		Probably not
		Definitely not
Q14	What are the primary barriers to adopting AI for implant success/failure assessment in your practice? (Select up to three)	Lack of training and education
		High cost of implementation
		Insufficient evidence on AI accuracy
		Concerns about reliability and errors
		Ethical and medico-legal concerns
		Lack of access to AI-integrated systems
		Resistance to change
		Concerns about AI replacing human roles
		Other (please specify): ______________
Q15	Would you support the inclusion of AI education in dental curricula and continuing professional development programs?	Strongly support
		Support
		Neutral
		Oppose
		Strongly oppose

## References

[1] Fahim YA, Hasani IW, Kabba S, Ragab WM (2025). Artificial intelligence in healthcare and medicine: clinical applications, therapeutic advances, and future perspectives. Eur J Med Res.

[2] Gao S, Wang X, Xia Z, Zhang H, Yu J, Yang F (2025). Artificial intelligence in dentistry: a narrative review of diagnostic and therapeutic applications. Med Sci Monit.

[3] Bansal RK, Arya A, Singh B, Singla M, Gupta S (2025). Role of artificial intelligence and machine learning in conservative dentistry and endodontics: a review. Cureus.

[4] Dilip Taide P, Faizan M, Salunkhe G, Sutradhar W, Yerra S, Gavara SG, Sharma M (2025). Automated prediction of dental implant success using a mask region-based convolutional neural network on preoperative cone-beam computed tomography scans. Cureus.

[5] Goey RS, Elenbaas L, Berkhout E (2025). AI acceptability in dentistry: Insights from dental professionals and students in the Netherlands: a pilot study. Int Dent J.

[6] Gupta S, Verma S, Chauhan AK, Roy MS, Rajkumari W, Sahgal C (2025). Knowledge, attitude, and perception of orthodontic students, and orthodontists regarding role of artificial intelligence in field of orthodontics-an online cross-sectional survey. J World Fed Orthod.

[7] Nouis SC, Uren V, Jariwala S (2025). Evaluating accountability, transparency, and bias in AI-assisted healthcare decision- making: a qualitative study of healthcare professionals' perspectives in the UK. BMC Med Ethics.

[8] Neji G, Gasparro R, Tlili M (2025). AI-powered predictive models in implant dentistry: planning, risk assessment, and outcomes. J Clin Med.

[9] Gan Y, Guo J, Zhai J, Huang J (2026). Application of artificial intelligence in education of prosthodontics and implant dentistry: a review. Adv Med Educ Pract.

[10] (2026). Sample size calculator. https://www.calculator.net/sample-size-calculator.html.

[11] Scheetz J, Rothschild P, McGuinness M (2021). A survey of clinicians on the use of artificial intelligence in ophthalmology, dermatology, radiology and radiation oncology. Sci Rep.

[12] Schwendicke F, Samek W, Krois J (2020). Artificial intelligence in dentistry: chances and challenges. J Dent Res.

[13] Banerjee M, Chiew D, Patel KT (2021). The impact of artificial intelligence on clinical education: perceptions of postgraduate trainee doctors in London (UK) and recommendations for trainers. BMC Med Educ.

[14] Liu H, Gong Z, Wen B (2026). Deep learning model development and clinical validation for radiographic surrogate markers of implant esthetic risk. NPJ Digit Med.

[15] Acerra A, Aliberti A, Amato A, Eccellente A, Santurro A, Giordano F (2026). Artificial intelligence in implant dentistry: clinical validity, diagnostic performance, surgical planning, and medico-legal implications - a narrative review. Dent J (Basel).

[16] Mugri MH (2025). Accuracy of artificial intelligence models in detecting peri-implant bone loss: a systematic review. Diagnostics (Basel).

[17] Nazari Y, Lngeroodi PF, Maddahi M, Kobravi S, Amin MR, Bargrizaneh AA, Fouladi S (2025). Artificial intelligence models and predicting implant success. Biomed Res Ther.

[18] Harish V, Morgado F, Stern AD, Das S (2021). Artificial intelligence and clinical decision making: the new nature of medical uncertainty. Acad Med.

[19] Rokhshad R, Ducret M, Chaurasia A (2023). Ethical considerations on artificial intelligence in dentistry: a framework and checklist. J Dent.

[20] Bajjad AA, Gupta S, Agarwal S, Pawar RA, Kothawade MU, Singh G (2024). Use of artificial intelligence in determination of bone age of the healthy individuals: a scoping review. J World Fed Orthod.

[21] Mintz Y, Brodie R (2019). Introduction to artificial intelligence in medicine. Minim Invasive Ther Allied Technol.

[22] Singh N, Pandey A, Tikku AP, Verma P, Singh BP (2023). Attitude, perception and barriers of dental professionals towards artificial intelligence. J Oral Biol Craniofac Res.

[23] Liu TY, Lee KH, Mukundan A, Karmakar R, Dhiman H, Wang HC (2025). AI in dentistry: Innovations, ethical considerations, and integration barriers. Bioengineering (Basel).

[24] Lee AT, Ramasamy RK, Subbarao A (2025). Understanding psychosocial barriers to healthcare technology adoption: a review of TAM Technology Acceptance Model and Unified Theory of Acceptance and use of technology and UTAUT frameworks. Healthcare (Basel).

